# Application of blended integrated revision course in clinical surgery in West Africa in response to Covid-19 pandemic: perception of trainee surgeons

**DOI:** 10.4314/ahs.v22i4.26

**Published:** 2022-12

**Authors:** Sebastian Okwuchukwu Ekenze, Enoch Ogbonnaya Uche, Ikenna Ifeanyi Nnabugwu, Vincent Enemuo, David Okoh, Uko Kalu Uko, Emmanuel Rapuluchukwu

**Affiliations:** Department of Surgery, Faculty of Medical Sciences, College of medicine, University of Nigeria, Enugu Campus

**Keywords:** Surgical training, COVID-19 pandemic, virtual training, blended format, West Africa

## Abstract

**Background/Objective:**

This study assessed the surgery residents' evaluation of blended delivery of the 2020 Integrated Revision Course in Clinical Surgery (IRCCS) of the West African College of Surgeons undertaken as a result of COVID-19 pandemic.

**Methods:**

We performed a cross-sectional survey of 234 participants of the 2020 IRCCS using self-administered questionnaire. The survey assessed the previous traditional course and various aspects of the novel blended course using 5-point Likert scale.

**Results:**

Overall, 186 (79.5%) responded. The blended course had overall mean rating of 4.92 (on a 5-point scale) compared to 4.05 for the previous traditional course. Of the virtual aspect of the blended course, didactic lectures had the best mean rating of 4.32, while unmanned OSCE had the least with mean rating of 3.30. Aspects of the multicentre component of the blended course were rated highly with conduct of manned OSCE receiving the best mean rating of 4.26. The major challenge of the blended course format was poor internet connectivity (n =102; 54.8%),

**Conclusion:**

Blended format of surgical training course is well rated by the surgery residents, and may be an effective means of delivery of clinical and non-clinical course contents during periods of disruption.

## Introduction

With the declaration of COVID-19 as a global pandemic by WHO in January 2020, the global healthcare delivery systems and educational systems were significantly hindered^1^, ^2^. In the field of surgery, the impact on surgical practice and training programmes led to changes in schedules; reduction in caseloads; efforts were expended to reduce unnecessary exposure to the virus; and a move to deliver surgery training curricula with virtual platforms to avoid large gathering^1^ - ^6^. Among the suggestions proffered was the use of a blended format combining traditional method with virtual method. Prior to the pandemic, a systematic review and meta-analysis^7^ demonstrated efficacy of the blended learning format in medical education. In the COVID-19 pandemic era some studies^1^, ^3^–^5^, ^8^ have looked at the application of blended format for surgical education. In these publications, though the trainees espoused the benefits of the blended format, they expressed reservations on the applicability of the format for assessments and the lack of face-to face clinical hands-on exposure. Hence, there is need for a variant of blended format of training that will incorporate a well-structured online teaching and assessment with multilocation structured onsite face-to face clinical hands-on teaching and assessment. The trainee perception and assessment of this format may highlight the potential applicability and benefits of the format in the future of surgical education during periods of disruption.

The West African College of Surgeons (WACS) organizes yearly integrated revision course for its trainees. During this 2-week course, the trainees are exposed to structured lectures on various aspects of surgery and surgical pathology. In addition, there are hands-on surgical instrumentation; clinical demonstration; Objective Structured Clinical Examination (OSCE) simulations; traditional clinical examination practice sessions; dissertation preparation and defence; and Computer Based Testing (CBT) practice examinations. In the preCOVID-19 era, these components were all integrated and undertaken onsite at a chosen accredited centre in the sub region.

Amidst the COVID-19 pandemic, the organizers of the 2020 course (Department of Surgery, University of Nigeria Teaching Hospital, Ituku-Ozalla, Enugu Nigeria) adopted a novel blended/hybrid approach to ensure full curriculum content delivery without breaching COVID-19 public health standards and policy guidelines. In this approach we organized the virtual rendering for the didactic lectures, surgical instrumentation, dissertation discussion, CBT and unmanned component of OSCE, surgical radiology and pathology demonstration, and multi-specialty tutorial panel. For the virtual component, we established an interactive website (www.wacscoacirccs. org) to facilitate organized content delivery. Other components of the course such as clinical demonstration, manned component of OSCE, and fellowship clinical examination were conducted on site at multiple training locations. For these multicentre manned activities, we selected twelves centres in the region based on proximity to the registered participant's training location, ensuring that no more than 15 participants were assigned to a selected centre with the aim of avoiding large gathering. The selected centres were prepped to simultaneously deliver the on-site activities which was coordinated by the organizing centre. Ahead of the commencement of the course, we organized multiple online training programmes to educate the both participants and resource faculty on the course delivery strategies. Feedback from these training programmes helped to refine the course delivery strategies.

This study assessed the surgery residents' evaluation of this novel blended delivery of the 2020 Integrated Revision Course in Clinical Surgery of the West African College of Surgeons and their outlook on the application of this format in future courses

## Materials and Methods

To evaluate the surgery residents' perceptions of this blended delivery of integrated revision course in clinical surgery, the participants attending the course were surveyed. Following ethical clearance by the hospitals' Health Research and Ethics Committee, the survey was administered virtually to the trainees via the course website. Before completing the survey, the respondents received a separate note detailing the voluntary nature of participation, the study procedure, risks, and confidentiality with regard to the information in the survey. Those who consented proceeded with the survey.

The survey instrument was a purpose designed questionnaire. The domains explored in the survey (Appendix 1) were 1) demographics, 2) assessment of aspects of the previous courses, 3) assessment of various aspects of the novel blended course including course preparations and delivery of the various components of the course, 4) challenges encountered in the blended delivery and their outlook on application of blended format in future courses.

The overall rating of the previous courses, and the assessment of the blended course preparation and the delivery of the course components were done quantitatively using appropriate 5-point Likert scale (1 very poor; 2 poor; 3 average; 4 good; 5 very good). The other responses were evaluated qualitatively. However, we used themes to group the responses from the qualitative data.

## Data analysis

Completed questionnaires were fed into a Statistical Package for Social Sciences (IBM SPSS Statistics Data Editor version 21) spreadsheet. This was used for data entry and analysis. Results were expressed as absolute values, percentages, median or mean.

## Results

Overall, 186 (79.5%) of the 234 participants completed the questionnaire. Of the respondents, 132 (71%) were registrars and 54 (29%) were senior registrars. There were 175 (94.1%) males and 11 (5.9%) females. Their median age was 35.5 years (range 27 – 53 years; Interquartile range IQR 33 – 38.3 years).

### Assessment of previous courses

A total of 114 (61.3%) of the respondents indicated attending previous WACS revision course organized in traditional format. The components of the course include pre-test, didactic lectures, tutorials, radiology and pathology demonstrations, surgical instrumentations, computer-based testing (CBT), hands-on clinical testing, dissertation demonstration, and interactive session on examinations. The mean rating for the previous course in terms of achieving the stated objectives by the 114 respondents was 4.05 (on a 5-point scale). Of these respondents, 37 (32.5%) rated the previous course as very good, while only 2 (1.7%) rated it as very poor.

In addition to the previous traditional course, 14 respondents also indicated attending a previous virtual course or training.

### Assessment of the novel blended course

The respondents indicated that the course poster and flyer was the most common medium through which they obtained information on the blended course (n = 142; 76.3%). The other media were WhatsApp (n = 84; 45.2%), WACS website (n = 78; 41.9%), course website (n = 46; 24.7%), Telegram (n = 10; 9.4%), Facebook (n = 3; 1.6%), and Twitter (n = 1; 0.5%). The mean rating of dissemination of information for this blended course was 4.40 (on a 5-point scale). A total of 181 (97.3%) respondents participated in the training and interaction organized to prepare them for this novel format of the course. The mean rating of the utility of this training/interaction was 4.61 (on a 5-point scale). On effectiveness of the course website, the aspect with the best rating was “accessibility of the course website” with a mean rating of 4.18 (on a 5-point scale). A summary of the ratings of information dissemination, utility of the training to prepare for the blended format, and effectiveness of the course website is shown in table-1.

**Table 1:** showing the rating of information dissemination, utility of the training for the blended format and effectiveness of the course website by the respondents

Aspect	Number of Participants	Rating (%)					Mean
		*Very poor*	*Poor*	*Average*	*Good*	*Very good*	Rating (in a 5-point scale
Information dissemination	186	0	1 (0.5)	15 (8.1)	78 (41.9)	92 (49.5)	4.40
Utility of training for blended Format	181	0	1 (0.5)	4 (2.2)	58 (32)	119 (65.7)	4.61
Accessibility of course website	186	7 (3.8)	2 (1.1)	26 (14)	67 (36)	84 (45.2)	4.18
Utility of course website	186	7 (3.8)	1 (0.5)	29 (15.6)	78 (41.9)	71 (38.2)	4.10
User friendliness of the course Website	186	7 (3.8)	2 (1.1)	37 (19.9)	66 (35.5)	74 (39.8)	4.06

The overall mean rating of the blended course in terms of achieving its stated objective was 4.92 (on a 5-pont scale). Table 2 shows comparison of the overall rating of the previous traditional course and the novel blended course.

**Table 2:** showing respondents' overall rating of the IRCCS course in traditional format and the blended format

Course	Number of Participants	Rating (%)					Mean
		*Very poor*	*Poor*	*Average*	*Good*	*Very good*	Rating (in a 5-point scale)
Previous (traditional format)	114	2 (1.7)	1 (0.8)	20 (17.5)	57 (50)	37 (32.5)	4.05
Current (blended format)	186	0	0	4 (2.2)	1 (0.5)	181 (97.3)	4.92

On the virtual components of the course 65 (39.4%) of the respondents rated the Pre-Course activities like sample CBT and OSCE demonstrations as very good. The mean rating of the various aspects of the virtual components of the course is shown in Table 3.

**Table 3:** showing the mean rating of the aspects of the virtual components of the course by 186 respondents

Aspects	Mean rating in a 5-point scale
Pre-Course CBT and OSCE	4.08
Pretest	4.09
Didactic lectures	4.32
Surgical instrumentation	4.20
Tutorials	4.20
Radiology and pathology demonstration	4.29
Dissertation preparation and defense	4.20
Computer-based testing (CBT)	4.12
Unmanned OSCE	3.30

The aspect of the multicentre hands-on clinical component of the course with the best rating was conduct of the manned OSCE (mean rating of 4.26 on a 5-point scale). Table 4 shows the rating of the various aspects of the multicentre hands-on clinical components.

**Table 4:** showing the rating of the various aspects of the multicentre hands-on clinical components by the respondents

Aspect	Number of Participants	Rating (%)					Mean
		*Very poor*	*Poor*	*Average*	*Good*	*Very good*	Rating (in a 5-point scale)
Notifications on the hands-on components	186	0	3 (1.6)	26 (14)	78 (41.9)	79 (42.5)	4.25
Adequacy of preparations at the centres	186	0	2 (1.1)	31 (16.7)	70 (37.6)	83 (44.6)	4.26
Conduct of the manned OSCE	132*	0	1 (0.8)	21 (15.9)	52 (39.4)	58 (43.9)	4.26
Conduct of the fellowship Clinicals	54#	0	1 (1.8)	9 (16.7)	23 (42.6)	21 (38.9)	4.21
Compliance with COVID-19 prevention strategies	186	1 (0.5)	2 (1.1)	31 (16.7)	69 (37.1)	83 (44.6)	4.24

* 132 Registrars participated in the manned OSCE

# 54 Senior Registrars participated in the fellowship clinicals

### Observed challenges of the blended format and the application in future courses

The most commonly cited challenge experienced by majority of the participants in the virtual aspect of the course was poor internet connectivity by 102 (54.8%), while the commonest challenge in the multicentre hands-on component was the stress of traveling to another centre from their base institution (n= 14; 7.5%). Summary of the observed challenges of the virtual and hands-on components as indicated by the respondents is shown in table 5. The respondents also indicated that the possible ways of addressing these challenges are by improving internet network connectivity (n = 60; 32.2%), improving resource persons' mastery of virtual platform use (n = 28; 15.1%), retooling the course website for easy navigation (n = 15; 8.1%), enhancing pre-course training (n = 13; 6.9%), providing access to video recording of lectures for downloads (n = 10; 5.4%), improving virtual platform control (n = 7; 3.8%), and improving feedback on performance at assessments (n = 7; 3.8%). A total of 46 (24.7%) respondents did not indicate possible solutions to the challenges.

**Table 5:** Summary of the challenges of the virtual and hands-on components indicated by the 186 respondents

Challenges	Number of respondents (%)
***Virtual components***	
Poor internet connectivity	102 (54.8%)
Website glitches during assessments	15 (8.1%)
Lack of mastery of virtual platform by resource persons	7 (3.8%)
Stress of 2-week course on virtual platform	3 (1.6%)
Cost of data	3 (1.6%)
Unstable electricity	3 (1.6%)
Inadequate virtual platform control	2 (1.1%)
None	51 (27.4%)
***Multicentre hands-on components***	
Stress of traveling to another centre	14 (7.5%)
Inadequate feedback on assessment	12 (6.5%)
Some centres were not well prepared	10 (5.4%)
None	150 (80.6%)

On future application of the blended structure, 108 of the 114 (94.7%) respondents that have experience of both format of the course suggested that the blended format be adopted for future rendering of IRCCS. The remaining 6 (5.3%) suggested that future IRCCS be conducted with the traditional format.

## Discussion

COVID-19 may be a defining global healthcare crisis with exceptional impact, but fortuitously, it may have provided the stimulus to try out blended rendering of this surgical training programme which may potentially offer limitless possibilities. Our survey has revealed that in the opinion of the surgical trainees in West Africa, some aspects of training and assessment in surgery which hitherto have been undertaken in traditional format with a lot of in-person interaction, can be carried out successfully with a blend of virtual and multicentre in-person arrangement. The respondents' opinion might be subjective, but these opinions have given insight into the prospects, processes, and challenges of this format of training.

The high overall rating of the blended format of training compared to the traditional format might indicate a preference for the blended format. Though similar high rating of blended format of training has been reported previously^8^, in interpreting this, there may be need for some considerations. Firstly, COVID is still evolving^4^, ^5^, ^8^, ^9^ and the blended format was introduced ostensibly to avoid large gatherings, hence cannot predict what may happen in the post COVID era with removal of restrictions on gatherings. Secondly, the virtual format of learning is an emerging experience that will require rigorous and regular evaluation to monitor its effectiveness^8^, ^10^. Despite these uncertainties, the COVID-19 pandemic has brought virtual training in surgery into a new light^8^, ^9^ and together with the novel blending to accommodate for hands-on training and assessment may help us substantially in post COVID era.

Of the virtual components of the training, didactic lectures and the other aspects of training requiring less in-person interactions received the highest rating. This might reflect a possible ease of delivery of these aspects of learning via virtual platform^2^, ^3^, ^5^, ^7^, ^8^,^10^. In addition to these aspects of learning, the virtual platform has been reported to also afford the potentials for use of real-time broadcast to teach and demonstrate common surgical procedures, demonstration of scenario of rare surgical disorders, improved interaction, real-time communication beyond classroom time-constraints, robust collaboration in training, and access to a network of geographically dispersed surgical experts^9^, ^11^ - ^13^. There is projection that the virtual learning platform is set to grow and expand in the post COVID era^9^, ^14^. The poor rating of virtual rendering of unmanned OSCE may reflects potential difficulty of adapting examinations and assessments in surgery via virtual platform. Previous studies^6^ - ^9^, ^14^ have highlighted the potential difficulty in transiting assessments in surgery which involves a lot of in-person interactions to a virtual activity. In these studies, the authors indicated that the feasibility of online assessment or a hybrid system for this was debatable. From the findings of the present study, it is evident that some aspects of assessments in surgery like picture tests, surgical cases scenarios, and surgical data interpretations which generally constitute the “unmanned OSCE” though rated poorly by the participants, can be conducted using virtual platforms. To improve on the assessment of these and standardize the testing using virtual platform, it may require adequate training and preparation of the participants, modification of the format, and retooling of the platform used.

There was relatively high rating of all the aspects of the multicentre components. These aspects represent the components of surgical training that require a lot of in-person interactions and conducting them in multicentre arrangement was akin to the traditional format. The fact that the multicentres were in the proximity of the participant's base and the limited number of participants per centre provided additional advantage that may endear this format for examination and assessments in surgery during pandemic periods or situations that warrant limitation of gatherings. This aspect may also be most suitable in a multi-regional setting where interregional transportation might be a challenge. Potential challenges might include personnel and financial cost of implementation of the programme, and difficulty with coordination.

The most important challenge with the blended system as the respondents indicated is related to internet access. This barrier to virtual learning reported from mostly resource-limited settings ranges from poor access and connectivity to lack of bandwidth and poor processing speed in mobile connections which leads to frequent disconnection^3^, ^13^, ^15^. The other challenges like defective power supply and cost of data are related to underdevelopment. Addressing these challenges will require concerted efforts to improve internet services and socioeconomic conditions of the trainees. Collaborations with more developed economies for provision of cheaper internet services, and the use of alternative electric power supply such as inverter or solar energy may boost these efforts^13^.

## Limitations of the study

Some aspects of blended course evaluated were not similar to what was evaluated for traditional course. Recall bias may influence objective evaluation of the previous course attended in the traditional format. These might have precluded objective evaluations, comparisons, and conclusions. Also, this was a self-assessment survey using instrument which is yet to be tested for reliability and consistency, and this introduced limitations and potential bias in the rating of the various aspects of the courses. In addition, mention was not made of the quality of delivery by the resource persons. This is important because the overall quality may be enhanced by better delivery by the resource persons. Finally, the qualitative data from the open questions was not coded and though we grouped the response from participants in themes, it opens up potential for reporting bias.

## Conclusion

This study has shown that in the West African setting, the trainee surgeons indicate that the use of blended format for delivery of surgical training course might be conducted successfully. This format was well rated by the trainees and may be an efficient means of delivering clinical and non-clinical course contents especially during periods of disruption. Despite this, there is need for rigorous and regular evaluation to monitor its efficacy. Effort geared towards improving internet connectivity and enhancing online testing protocol may further strengthen this format for future of surgical education.

## References

[1] Aziz H, James T, Remulla D, Sher L, Genyk Y, Sullivan ME (2021). Effect of COVID-19 on Surgical Training Across the United States: A National Survey of General Surgery Residents. J Surg Educ.

[2] Doulias T, Gallo G, Rubio-Perez I, Breukink SO, Hahnloser D (2020). Doing More with Less: Surgical Training in the COVID-19 Era. J Invest Surg.

[3] Chandrasinghe PC, Siriwardana RC, Kumarage SK, Munasinghe BNL, Weerasuriya A, Tillakaratne S (2020). A novel structure for online surgical undergraduate teaching during the COVID-19 pandemic. BMC Med Educ.

[4] Lerendegui L, Boudou R, Percul C, Curiel A, Durante E, Moldes JM (2021). Impact of the COVID-19 pandemic on surgical skills training in pediatric surgery residents. Pediatr Surg Int.

[5] Ellison EC, Nagler A, Stain SC, Matthews JB, Spanknebel K, Shabahang MM (2021). Impact of the COVID-19 pandemic on surgical trainee education and well-being spring 2020-winter 2020: A path forward. Am J Surg.

[6] Ellis R, Scrimgeour DSG, Brennan PA (2020). Surgical training during the COVID-19 pandemic: preparing for future uncertainty. Br J Oral Maxillofac Surg.

[7] Vallée A, Blacher J, Cariou A, Sorbets E (2020). Blended Learning Compared to Traditional Learning in Medical Education: Systematic Review and Meta-Analysis. J Med Internet Res.

[8] Rimsha S, Moosa FA, Zaheer F, Kamal MT, Majid A (2021). What Does the Future Hold for a Surgical Trainee? This Lockdown Is Not a Letdown Yet: A Survey on Moodle Learning Management System as a Part of Blended Learning During COVID-19 Pandemic. Cureus.

[9] Ngoo KS, Fadzli AN, Amponin MOCSE, Cho SY, Chuang Y, Horiguchi A (2021). COVID-19 pandemic impact on urology residencies in Asia - An observational study. Surg Pract.

[10] Khalil R, Mansour AE, Fadda WA, Almisnid K, Aldamegh M, Al-Nafeesah A (2020). The sudden transition to synchronized online learning during the COVID-19 pandemic in Saudi Arabia: a qualitative study exploring medical students' perspectives. BMC Med Educ.

[11] Bhatti I, Jones K, Richardson L, Foreman D, Lund J, Tierney G (2011). E-learning vs lecture: which is the best approach to surgical teaching?. Colorectal Dis.

[12] Jayakumar N, Brunckhorst O, Dasgupta P, Khan MS, Ahmed K (2015). e-Learning in surgical education: a systematic review. J Surg Educ.

[13] Ekenze SO, Okafor CI, Ekenze OS, Nwosu JN, Ezepue UF (2017). The Value of Internet Tools in Undergraduate Surgical Education: Perspective of Medical Students in a Developing Country. World J Surg.

[14] English W, Vulliamy P, Banerjee S, Arya S (2020). Surgical training during the COVID-19 pandemic - the cloud with a silver lining?. Br J Surg.

[15] Neupane HC, Sharma K, Joshi A (2020). Readiness for the Online Classes during COVID-19 Pandemic among Students of Chitwan Medical College. J Nepal Health Res Counc.

